# Investigation into the Effects of Tramadol, Citalopram, Tianeptine, and Their Combinations on Rat Brain Tissue

**DOI:** 10.3390/biomedicines13071690

**Published:** 2025-07-10

**Authors:** Irem Ates, Bahar Isik, Fusun Gozen, Gulce Naz Yazici, Mine Gulaboglu, Renad Mammadov, Gulbeniz Huseynova, Durdu Altuner, Halis Suleyman

**Affiliations:** 1 Department of Anesthesiology and Reanimation, Faculty of Medicine, Ataturk University, Erzurum 25240, Turkey; driremates@hotmail.com; 2 Department of Emergency Medicine, Faculty of Medicine, Ataturk University, Erzurum 25240, Turkey; drbaharisik7@gmail.com; 3 Department of Anesthesiology and Reanimation, Bursa High Specialization Training and Research Hospital, University of Health Sciences, Bursa 16310, Turkey; fusungozen@gmail.com; 4 Department of Histology, Faculty of Medicine, Erzincan Binali Yıldırım University, Erzincan 24100, Turkey; gulcenazyazici.ank@gmail.com; 5 Department of Biochemistry, Faculty of Pharmacy, Ataturk University, Erzurum 25240, Turkey; minegulaboglu@atauni.edu.tr; 6 Department of Pharmacology, Faculty of Medicine, Erzincan Binali Yıldırım University, Erzincan 24100, Turkey; mammadovrenad24@gmail.com (R.M.); durdualtuner@hotmail.com (D.A.); 7 Department of Pharmacology, Azerbaijan Medical University, Baku AZ1022, Azerbaijan; huseynovagulbaniz04@gmail.com

**Keywords:** tramadol, citalopram, tianeptine, rat, brain

## Abstract

**Background**: Tramadol binds to opioid receptors and inhibits norepinephrine and serotonin reuptake, causing serotonin syndrome. Tianeptine stimulates serotonin reuptake and reduces serotonin levels. The aim of this study was to investigate whether tianeptine is effective against serotonin syndrome that may occur with serotoninergic drugs such as tramadol and citalopram. **Methods**: Rats were divided into eight groups (n = 6) that received tramadol (50 mg/kg), citalopram (10 mg/kg), or tianeptine (5 mg/kg) alone or a combination of tramadol + citalopram, tramadol + tianeptine, citalopram + tianeptine or tramadol + citalopram + tianeptine at the same doses administered to the stomach by oral gavage for 3 weeks. The healthy control group was given saline. Malondialdehyde, total glutathione, superoxide dismutase, and catalase levels were measured in removed brain tissues. The tissues were also examined histopathologically. **Results**: In the tramadol, tramadol + citalopram, and tramadol + citalopram + tianeptine groups, malondialdehyde levels were found to be higher compared to the control group, while glutathione, superoxide dismutase, and catalase levels were found to be lower. In other groups, values close to the control group were measured. Morphological degeneration was observed in neurons in the tramadol + citalopram group. The swelling of astrocytes and pericellular edema in oligodendrocytes were also observed. A significant population increase was noted in microglial cells. Blood vessels belonging to the tissue were observed to be severely dilated and congested. Histopathological damage was partially resolved in the group given tramadol + citalopram + tianeptine. **Conclusions**: The tramadol + citalopram combination caused severe oxidative stress in brain tissue. Tramadol alone caused mild damage in brain tissue, whereas tianeptine prevented the brain damage caused by tramadol.

## 1. Introduction

Tramadol has been approved by the U.S. Food and Drug Administration (FDA) specifically for the treatment of moderate to severe pain [1]. Tramadol is a centrally acting opioid analgesic that binds to μ-opioid receptors and inhibits the reuptake of norepinephrine and serotonin [2]. Currently, in cases of uncontrolled pain, analgesics are administered in repeated or high doses. However, with increasing doses of tramadol, the level of malondialdehyde (MDA), an oxidative parameter in the brain, increases proportionally, while the levels of endogenous antioxidants such as glutathione (GSH), superoxide dismutase (SOD), and catalase (CAT) decrease [3]. Additionally, the inhibition of serotonin reuptake by tramadol leads to side effects. This toxicity, known as serotonin syndrome, occurs as a result of tramadol overdose or misuse [4]. In untreated patients, these side effects can result in high morbidity and mortality rates [5]. Serotonin syndrome, in particular, is a potentially life-threatening adverse drug reaction caused by drug interactions [6]. It often develops after the administration of another drug that increases serotonin levels in patients already receiving a serotonergic drug [7]. Tramadol has a high risk of causing serotonin syndrome when used in combination with serotonergic antidepressants [8]. In the literature, a typical case of serotonin syndrome associated with the combination of tramadol and citalopram has been reported [9]. Citalopram is a selective serotonin reuptake inhibitor (SSRI). Citalopram hydrobromide exerts its antidepressant effect by enhancing serotonergic activity in the central nervous system [10]. Reports have indicated that the combination therapy of tramadol with selective serotonin reuptake inhibitors (SSRIs) such as fluoxetine, paroxetine, sertraline, and citalopram may cause serotonin syndrome [9].

Tianeptine is an atypical antidepressant structurally resembling tricyclic antidepressants [11]. It is considered unique among antidepressants as its mechanism of action appears to be independent of serotonin receptors and monoamine transport activity [12]. Tianeptine is known to be favored in the treatment of resistant depression [13]. Unlike many other antidepressants, tianeptine has been reported to stimulate serotonin reuptake and consequently decrease serotonin levels [14,15]. Previous studies have also demonstrated that tianeptine exerts antioxidant effects. Its antioxidant action is thought to be associated with the suppression of MDA elevation and the prevention of decreases in the activities of antioxidant enzymes such as CAT and SOD [16]. Tianeptine’s ability to reduce serotonin syndrome may be due to its ability to reduce serotonin levels [17].

The information obtained from the literature suggests that the combined use of tramadol with SSRIs may lead to serotonin toxicity. It also indicates that tianeptine might be effective against serotonin syndrome induced by serotonergic agents. However, no information was found in the literature regarding the effects of tianeptine on serotonin syndrome caused by the use of serotonergic drugs. The aim of our study was to investigate the effects of tramadol, citalopram, tianeptine, and their combinations on brain tissue.

## 2. Materials and Methods

### 2.1. Animals

The animals were divided into groups before the experiment and fed animal feed under appropriate conditions in an environment with a 12 h light and 12 h dark cycle at normal room temperature (22 °C). The protocols and procedures were approved by the local Animal Experimentation Ethics Committee (date: 27 February 2025; meeting no: 2025/02)

### 2.2. Chemicals

Thiopental sodium used in this study was supplied by IE Ulagay Pharmaceutical Co. (Istanbul, Türkiye), tramadol HCl and citalopram were supplied by Abdi İbrahim Pharmaceutical Industry (Istanbul, Türkiye), and tianeptine was supplied by Servier Pharmaceutical Co. (Istanbul, Türkiye).

### 2.3. Experimental Groups

The animals were divided into eight different groups as follows: healthy control (HC), tramadol alone (T), citalopram alone (C), tianeptine alone (Ty), tramadol + citalopram (T + C), tramadol + tianeptine (T + Ty), citalopram + tianeptine (C + Ty), and tramadol + tianeptine + citalopram (T + Ty + C).

### 2.4. Experimental Procedure

The animals in the T (n = 6), C (n = 6), and Ty (n = 6) groups received tramadol (50 mg/kg), citalopram (10 mg/kg), and tianeptine (5 mg/kg), respectively, administered orally via gavage. In a previous study, tramadol administered to animals at a dose of 50 mg/kg was shown to induce oxidative stress [18]. Additionally, it has been demonstrated that citalopram at a dose of 10 mg/kg improves depression-like behaviors in rats more effectively than the 5 mg/kg dose [19]. Moreover, tianeptine has been reported to exert antioxidant and protective effects at a dosage of 5 mg/kg [20]. In the T + C (n = 6) group, tramadol + citalopram; in the T + Ty (n = 6) group, tramadol + tianeptine; in the C + TY (n = 6) group, citalopram + tianeptine; and in the T + Ty + C (n = 6) group, tramadol + citalopram + tianeptine were administered at the specified doses using the same oral gavage method. The HC (n = 6) group was given distilled water orally as a solvent using the same method. This procedure was repeated once daily for three weeks. During this time, the animals were observed for any behavioral changes or abnormalities. At the end of this period, the animals were euthanized with high-dose thiopental sodium (50 mg/kg) anesthesia and their brain tissues were removed. Levels of MDA, tGSH, (SOD) and CAT were measured in the removed tissues. Tissues were also examined histopathologically. The results obtained from all experimental groups were evaluated by comparison between the groups.

### 2.5. Biochemical Analysis

#### 2.5.1. Preparation of Samples

The brain tissue samples obtained from the animals were first washed in physiological solution. Then, the tissues were pulverized under liquid nitrogen and then homogenized. The obtained supernatants were used for the analyses of MDA, GSH, SOD, and CAT.

#### 2.5.2. Determination of MDA, GSH, SOD, and CAT in Brain Tissue

The levels of SOD, GSH, and MDA in brain tissues were measured using rat ELISA kits following the instructions provided by the manufacturers (Catalog numbers: 706002, 703002, and 10009055, respectively; Cayman Chemical Company, Ann Arbor, MI, USA). The CAT level was determined using the method described by Goth [21].

### 2.6. Histopathological Examinations

The tissues of the subjects were fixed for 72 h in 10% formaldehyde solution. After the fixation process, the tissues were taken in a cassette and washed in running water for 24 h. They were then purified from water by passing through increasing alcohol series (70%, 80%, 90%, and 100%). The brain tissues, which were made transparent in xylene, were embedded in paraffin blocks, and 4–5-micron-thick sections were taken. The sections were stained with hematoxylin–eosin dual staining and evaluated and photographed in the Olympus DP2-SAL firmware program (Olympus^®^ Inc. Tokyo, Japan). Histopathological evaluation was performed by a histologist who was blind to the study groups. All histopathological findings were evaluated semi-quantitatively as none (0), mild (1), moderate (2), and severe (3).

### 2.7. Statistical Analyses

The results obtained from the experiments were expressed as “mean value ± standard error of mean” (x ± SEM). The significance level of the difference between the groups was determined using the one-way ANOVA test. A post hoc Tukey test was performed afterwards. A Kruskal–Wallis test was performed for nonparametric data. All statistical analyses were performed using the “SPSS for Windows (version 18.0; IBM Corporation, Armonk, NY, USA, 2018)” statistics program, and *p* < 0.05 was considered significant.

## 3. Results

### 3.1. Behavioral Test Results

In animals belonging to the T + C group, pronounced hyperactivity was evident, especially upon opening the cage lid. Jump responses were triggered during physical interactions between animals. During rest, apart from hind limb abduction and a low body posture, no further behavioral anomalies were observed. In the T + Ty + C group, pronounced hyperactivity was the only notable behavioral finding. No signs indicative of behavioral impairments were identified in the other experimental groups.

### 3.2. Biochemical Findings

#### 3.2.1. MDA Analysis Results of Brain Tissue

**Figure 1:**
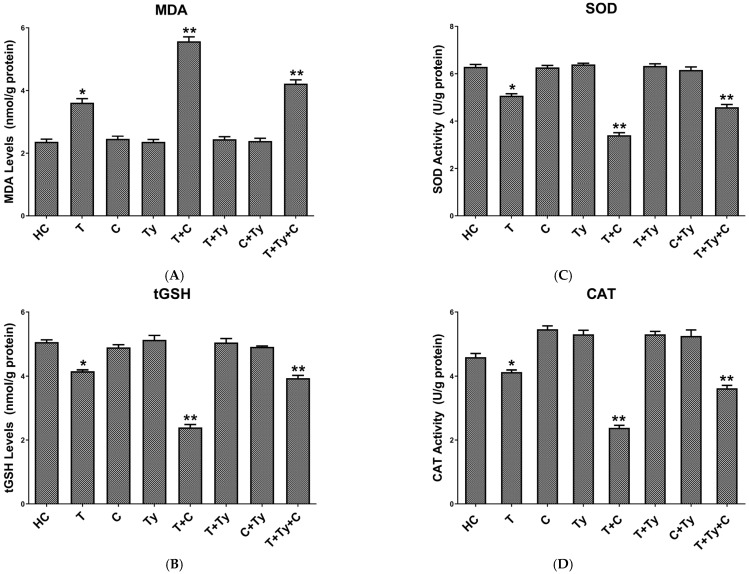
(**A**) MDA, (**B**) tGSH, (**C**) SOD, and (**D**) CAT levels of study groups. * *p* < 0.05 according to HC group; ** *p* < 0.0001 according to HC group. MDA: malondialdehyde; tGSH: total glutathione; SOD: superoxide dismutase; CAT: catalase; HC: healthy control; T: tramadol alone; C: citalopram alone; Ty: tianeptine alone; T + C: tramadol + citalopram; T + Ty: tramadol + tianeptine; C + Ty: citalopram + tianeptine; T + Ty + C: tramadol + tianeptine + citalopram.

As shown in Figure 1A, the MDA level in the brain tissue of animals treated with tramadol alone was significantly increased compared to the healthy control group (*p* < 0.05). The highest MDA level was observed in the group receiving the tramadol + citalopram combination (T + C) when compared to the healthy group (*p* < 0.0001). In the group treated with the tramadol + citalopram + tianeptine combination (T + Ty + C), the MDA level was also significantly elevated compared to the healthy control group (*p* < 0.0001). In contrast, MDA levels in the groups treated with citalopram (C), tianeptine (Ty), tramadol + tianeptine (T + Ty), and citalopram + tianeptine (C + Ty) combinations were found to be similar to be close to the healthy group, with no statistically significant differences (*p* > 0.05).

#### 3.2.2. Brain Tissue tGSH Analysis Results

As shown in Figure 1B, the tGSH level in the brain tissue of animals in the T group was significantly lower than the healthy control group (*p* < 0.05). The lowest tGSH level was observed in the T + C group compared to the healthy group (*p* < 0.0001). In the T + Ty + C group, which received the triple combination treatment, the tGSH level was also significantly reduced compared to the healthy group (*p* < 0.0001). However, in the C, Ty, T + Ty, and C + Ty groups, the tGSH levels were found to be close to those of the healthy group, with no statistically significant differences observed (*p* > 0.05).

#### 3.2.3. Brain Tissue SOD Analysis Results

In our study, the SOD activity in the brain tissue of animals in the T group was significantly decreased compared to the healthy control group (*p* < 0.05). In the C and Ty groups, which received citalopram and tianeptine alone, respectively, the SOD activity levels were not significantly different from those of the healthy group (*p* > 0.05). Likewise, no significant changes in SOD activity were observed in the T + Ty and C + Ty groups (*p* > 0.05). On the other hand, in the T + C and T + Ty + C groups (*p* < 0.0001), SOD activity was found to be significantly lower than in the healthy control group (Figure 1C).

#### 3.2.4. Brain Tissue CAT Analysis Results

As seen in Figure 1D, the CAT activity levels in the brain tissue of animals in the C, Ty, T + Ty, and C + Ty groups were found to be very close to those of the healthy group, and the differences between them were not statistically significant (*p* > 0.05). In the T group, SOD activity was found to be slightly lower than that of the healthy group (*p* < 0.05), while a more significant decrease was observed in the T + Ty + C and the T + C groups (*p* < 0.0001).

### 3.3. Histopathological Findings of Brain Tissue

**Figure 2:**
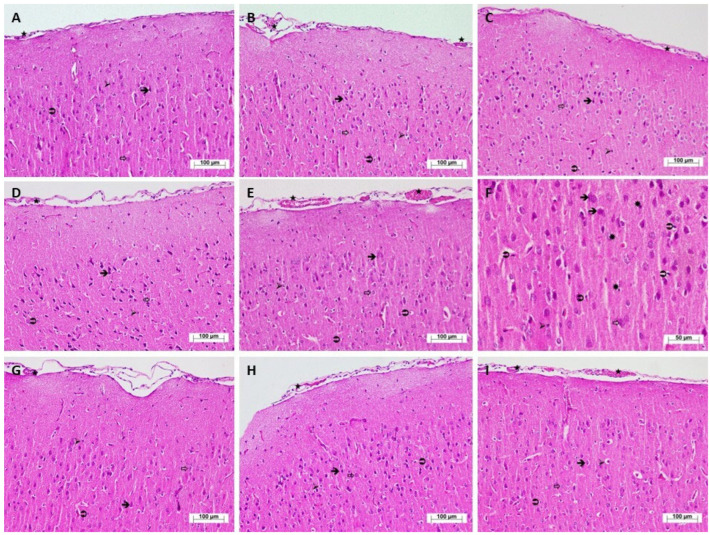
Hematoxylin–eosin-stained histopathological appearance of cerebral cortex tissues of the study groups. (**A**) Cerebral cortex tissue of HC group rats (H&E, ×200). (**B**) Cerebral cortex tissue of T group rats (H&E, ×200). (**C**) Cerebral cortex tissue of C group rats (H&E, ×200). (**D**) Cerebral cortex tissue of Ty group rats (H&E, ×200). (**E**) Cerebral cortex tissue of T + C group rats (H&E, ×200). (**F**) Cerebral cortex tissue of T + C group rats (H&E, ×400). (**G**) Cerebral cortex tissue of T + Ty group rats (H&E, ×200). (**H**) Cerebral cortex tissue of C + Ty group rats (H&E, ×200). (**I**) Cerebral cortex tissue of T + Ty + C group rats (H&E, ×200). 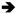: neuron (**A**–**D**,**G**–**I**), degenerated neuron (**E**,**F**); ⇒: astrocyte (**A**–**D**,**G**–**I**), swollen astrocyte (**E**,**F**); ➢: oligodendrocyte (**A**,**C**,**D**,**G**,**H**), slightly edematous oligodendrocyte (**B**), pericellular edema oligodendrocyte (**E**,**F**), moderately edematous oligodendrocyte (**I**); 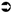: microglia (**A**–**D**,**G**–**I**), increased microglia (**E**,**F**); ★: blood vessel (**A**,**C**,**D**,**G**,**H**), moderately dilated and congested blood vessel (**B**,**I**), severely dilated and congested blood vessel (**E**); ✹: apoptotic body (**F**).

As seen in Figure 2A, the histopathological evaluation of cerebral cortex tissue sections from the HC group revealed that the neurons, as well as astrocytes, which are supporting cells of the nervous tissue, oligodendrocytes, and microglia, were observed to have a normal structure and distribution. In the T group, which received tramadol alone, the neurons generally exhibited normal morphology. While astrocytes mostly maintained their typical structural appearance, mild edema was observed in oligodendrocytes, and microglial cells demonstrated a normal distribution. There was also evidence of moderate vascular dilation and congestion (Figure 2B). In the C group, which received citalopram alone, the histological appearance was similar to that of the control group (Figure 2C). The Ty group, administered tianeptine alone, exhibited a normal histopathological profile (Figure 2D). In the T + C group, which received the combination of tramadol and citalopram, morphological degeneration was noted in neurons. Astrocytic swelling and pericellular edema in oligodendrocytes were additionally observed. Compared to the control group, there was a marked increase in the population of microglial cells. Blood vessels within the tissue were found to be severely dilated and congested (Figure 2E). Furthermore, the examination of high-magnification sections from this group revealed the presence of apoptotic bodies within the nervous tissue in addition to other histopathological findings (Figure 2F). In the brain tissue of the T + Ty group (tramadol and tianeptine combination) and the C + Ty group (citalopram and tianeptine combination), normal histopathological findings were observed (Figure 2G,H). In the T + Ty + C group, which received the triple combination of tramadol, citalopram, and tianeptine, neurons and astrocytes exhibited normal distribution and morphology; however, oligodendrocytes showed moderate edema in certain regions. Microglial cells demonstrated a normal distribution while vascular structures showed moderate dilation and congestion (Figure 2I). The evaluation of the grades of histopathological damage is shown in Table 1.

## 4. Discussion

In this study, the effects of tramadol, citalopram, tianeptine, and their combinations on brain tissue were investigated. As discussed above, the concurrent use of selective serotonin reuptake inhibitors (SSRIs) can lead to a condition known as serotonin syndrome, which is a form of drug-induced toxicity [7]. Serotonin syndrome is a rare but potentially life-threatening adverse effect resulting from elevated serotonin levels [22]. In untreated patients, these adverse effects may result in high morbidity and mortality [5]. According to the literature, the combined use of tramadol with citalopram or other SSRIs has been associated with the development of serotonin syndrome [9]. Moreover, serotonin syndrome has also been observed in cases of tramadol overdose or prolonged misuse when used alone [4]. In our study, the potential neurotoxic effect of tramadol on brain tissue was evaluated by measuring oxidant and antioxidant parameter levels. Our experimental results demonstrated that MDA levels, which reflect oxidative stress, were elevated in the brain tissue of animals treated with tramadol. MDA is the end product of membrane lipid peroxidation (LPO) during the oxidative stress process [23]. 

Previous studies have indicated that the formation of reactive oxygen species (ROS) plays a role in many diseases [24]. In particular, their role in the pathogenesis of neuropsychiatric disorders has attracted attention [25]. It is commonly recognized that MDA measurement provides a crucial marker for detecting ROS damage [26]. Neurodegenerative cellular damage occurring in the brain during tramadol abuse or intoxication has been associated with oxidative stress [27]. In their study, Hussein and colleagues (2020) showed that tramadol administration increased MDA levels in rat brain tissue, reduced antioxidant levels, and caused damage in the brain due to oxidative stress [28].

According to the literature, GSH, SOD, and CAT are known to be among the key endogenous antioxidant defense mechanisms [29]. In tramadol-treated animals, the decrease in antioxidant enzyme levels (tGSH, SOD, and CAT) accompanying the increase in MDA supports the findings previously reported by Ali and colleagues [30]. In a study conducted by Mohammadnejad and Soltaninejad (2022), the decrease in SOD and CAT activities and the increase in MDA levels were associated with oxidative stress induced by tramadol toxicity [31]. Our experimental findings indicate that tramadol induces a significant level of oxidative stress in brain tissue.

In the brain tissues of animals administered citalopram alone, no significant changes were observed in the levels of MDA, tGSH, SOD, and CAT, indicating the absence of oxidative stress. However, the combination of citalopram and tramadol altered the oxidant-antioxidant balance in the brain tissue of the animals to levels indicative of oxidative stress. Mikkelsen and colleagues (2023) stated that serotonergic drugs, whether used at therapeutic or overdose levels or in combination, may lead to serotonin syndrome [32]. The behavioral disturbances observed in the animal groups treated with dual serotonergic drug combinations suggest that our experimental findings are in concordance with the existing literature. As mentioned above, serotonin syndrome is a side effect caused by increased serotonin levels [22].

As serotonin levels increase, the production of ROS also rises, and the antioxidant defense systems become insufficient [25]. Another study found that SSRIs inhibit the CYP450 and CYP2D6 enzymes that play a role in tramadol metabolism, leading to tramadol accumulation and enhanced serotonergic activity [33]. This condition, resulting from the concurrent use of tramadol and SSRIs, is frequently discussed in the literature [34,35]. It is also argued that serotonin can be converted into toxic metabolites and may contribute to the development of neurodegenerative diseases [36].

In our study, no significant changes were observed in the oxidant and antioxidant levels in the brain tissue of animals treated with tianeptine alone. Interestingly, tianeptine significantly prevented the oxidative stress induced by tramadol in brain tissue. Moreover, in the tramadol + citalopram + tianeptine triple combination group, significant changes were observed between the oxidant and antioxidant levels in the brain tissue. This indicates that tianeptine significantly inhibited the increase in oxidant levels and the decrease in antioxidant levels induced by tramadol and citalopram. In the study by Della and colleagues (2012), it was emphasized that tianeptine, which improves stress-related cognitive impairment, may alleviate oxidative stress by increasing SOD and CAT activities in an animal model [37]. Previous studies have reported that the antioxidant effect of tianeptine is due to its ability to suppress the increase in MDA levels and the decrease in the activities of antioxidant agents such as CAT and SOD [16].

The literature also notes that tianeptine stimulates serotonin reuptake and reduces serotonin levels [14,15]. These findings suggest that tianeptine exerts its antioxidant effects by reducing serotonin levels. The view that serotonin can be converted into toxic metabolites and may contribute to the development of neurodegenerative diseases [36] is consistent with our experimental findings. It has been reported that tianeptine’s ability to reduce serotonin syndrome is most likely due to its effect in lowering serotonin levels [17].

Our experimental results evidence that the biochemical findings are in concordance with the histopathological observations. The severity of histopathological damage exhibited a parallel relationship with the elevation of oxidant parameters and the reduction in antioxidant levels. Our histopathological findings demonstrated that mild damage occurred in the brain tissue of animals treated with tramadol, accompanied by an increase in serotonin levels. Slettedal and colleagues (2011), in their study involving the administration of certain SSRIs and other antidepressants, reported the loss of Purkinje and granule cells in the brain tissue of subjects diagnosed with serotonin syndrome [38]. Another study suggested that SSRIs may lead to myelin malformation and oligodendrocyte pathology [39]. Furthermore, SSRIs have been shown to induce the formation of ROS, mitochondrial damage, and astrocyte apoptosis, which may contribute to the pathogenesis of neurodegenerative diseases [40].

## 5. Conclusions

As stated in the literature, serotonin syndrome arises from the concomitant use of SSRIs and other serotonergic agents. In line with previous reports, the present study demonstrated that the combination of tramadol and citalopram induced severe oxidative stress in brain tissue. According to our findings, tramadol alone caused mild damage in the brain. Neither citalopram nor tianeptine, when administered alone or in combination, resulted in detectable brain damage. Moreover, tianeptine was found to prevent tramadol-induced neurotoxicity. In the group receiving the tramadol + citalopram + tianeptine combination, moderate brain damage was observed. The fact that tianeptine does not cause damage in the brain and prevents the damage induced by tramadol may be attributed to its enhancement of serotonin reuptake at synapses. Our experimental results suggest that tianeptine may be beneficial in mitigating the potential neurotoxic effects of serotonergic agents on brain tissue. To partially elucidate the mechanisms underlying the effects of tramadol, citalopram, tianeptine, and their combinations on brain tissue, it may be beneficial to measure blood serotonin levels, evaluate whether increased oxidative stress and decreased antioxidant capacity are associated with proinflammatory cytokine levels, assess DNA oxidation damage markers, and conduct immunohistochemical analyses of brain tissue.

## Figures and Tables

**Table 1:** Evaluation of histopathological findings of the study groups.

Groups	Astrocytes Median (Min-Max)	Oligodendrocytes Median (Min-Max)	Microglia Median (Min-Max)	Vascular Dilatation Median (Min-Max)	Congestion Median (Min-Max)	Apoptotic Bodies Median (Min-Max)
HC	0 (0–0)	0 (0–0)	0 (0–0)	0 (0–0)	0 (0–0)	0 (0–0)
T	0 (0–1)	1 (0–2)	0 (0–0)	2 (1–3)	2 (2–2) *	0 (0–0)
C	0 (0–0)	0 (0–0)	0 (0–0)	0 (0–0)	0 (0–0)	0 (0–0)
Ty	0 (0–0)	0 (0–0)	0 (0–0)	0 (0–0)	0 (0–0)	0 (0–0)
T + C	3 (2–3) *	3 (2–3) **	3 (1–3) *	3 (3–3) *	3 (2–3) *	1.5 (1–2) **
T + Ty	0 (0–0)	0 (0–0)	0 (0–0)	0 (0–0)	0 (0–0)	0 (0–0)
C + Ty	0 (0–0)	0 (0–0)	0 (0–0)	0 (0–0)	0 (0–0)	0 (0–0)
T + Ty + C	0 (0–0)	2 (1–3) **	0 (0–0)	2 (1–3) *	2 (1–2)	0 (0–0)

* *p* < 0.0001, according to HC group. ** *p* < 0.05, according to HC group.

## Data Availability

The original contributions presented in this study are included in the article. Further inquiries can be directed to the corresponding author.

## Abbreviations

The following abbreviations are used in this manuscript:

FDA	Food and Drug Administration
MDA	malondialdehyde
GSH	glutathione
SOD	superoxide dismutase
CAT	catalase
SSRI	selective serotonin reuptake inhibitor
HC	healthy control
T	tramadol alone
C	citalopram alone
Ty	tianeptine alone
T + C	tramadol + citalopram
T + Ty	tramadol + tianeptine
C + Ty	citalopram + tianeptine
T + Ty + C	tramadol + tianeptine + citalopram
x ± SEM	mean value ± standard error of mean
ANOVA	analysis of variance
LPO	lipid peroxidation
ROS	reactive oxygen species
